# Re-centering labour in local food: local washing and the growing reliance on permanently temporary migrant farmworkers in Nova Scotia

**DOI:** 10.1007/s10460-022-10397-0

**Published:** 2022-11-21

**Authors:** Elizabeth Fitting, Catherine Bryan, Karen Foster, Jason W. M. Ellsworth

**Affiliations:** 1 Department of Sociology and Social Anthropology, Dalhousie University, 6135 University Avenue, Halifax, NS B3H 4R2 Canada; 2 School of Social Work, Dalhousie University, 1459 LeMarchant Street, Halifax, NS B3H 4R2 Canada

**Keywords:** Local washing, Migrant farmworkers, Farmers’ markets, Labour, Alternative food initiatives, Canada, Seasonal Agricultural Workers Program (SAWP)

## Abstract

This article explores the labour behind local food in the Canadian Atlantic province of Nova Scotia. Based on surveys and interviews with farmers, migrant farmworkers, and farmers’ market consumers in the province, we suggest that the celebration of local food by government and industry is a form of “local washing.” Local washing hides key aspects of the social relations of production: in this case, it hides insufficient financial and policy supports for Nova Scotian farms and the increased reliance on migrant farmworkers via the Seasonal Agricultural Worker Program and the Temporary Foreign Worker Program. Our research found that a growing reliance on migrant farmworkers was not just the case for larger, industrial farms, but also for smaller farms participating in local and alternative food initiatives, like farmers’ markets and fresh produce subscription boxes. Additionally, our surveys show that while farmers’ market shoppers expressed an interest in supporting local foods, they reported knowing little about farm workers or working conditions. Our paper contributes to the literature on local and alternative food initiatives by connecting the relations of production to consumption. Rather than focusing solely on the nature of the relationships between farmers and consumers and the values embodied in direct agricultural markets, this research explores the central role of permanently temporary migrant workers in local agriculture.

## Introduction

Efforts to re-localize food and support local food production have gained popularity in North America and elsewhere, as producers, consumers, and policy makers are increasingly reflexive about food and eating, due in part to the growing awareness of the deleterious effects of the conventional food system on the climate, the environment, human health and wellbeing, animal welfare, and livelihoods. More recently, the global COVID-19 pandemic—which caused disruptions and delays in the food chain—has highlighted the importance, and precarity, of the work of growing, processing, packaging, transporting, and distributing the world’s food.

An increasing number of farms rely on precarious migrant labour in many parts of the world, including in the Canadian province of Nova Scotia, where our research is based. Located on Canada’s east coast, Nova Scotia has a unique and long-standing reputation for food localization efforts, yet over the past twenty years, the reliance on migrant labour has risen, including on farms that participate in local food initiatives and direct agricultural markets like farmers’ markets and fresh produce subscription boxes. While the labour behind food production and processing is examined in various literatures—especially those focused on commodity food chains, the political economy of food, and labour migration studies—migrant labour remains understudied in alternative agriculture, localization initiatives and direct agricultural markets, with some notable exceptions (see, for example, Gray 2014; Guthman 2004; Weiler et al. 2016).

Based on two years of research in Nova Scotia, we add to this literature by bringing migrant labour into an analysis of local^1^ agriculture and by exploring the perspectives of farmers, farmworkers *and* consumers who participate in direct agricultural markets. We find a tension between the province’s stated commitments to strengthening local food consumption and production and what actually happens: although local food is proudly marketed in Nova Scotia and successive provincial governments have set policy goals to bolster local food production and consumption, those goals have not been met, the consumption of locally produced food remains low,^2^ and there has been an increased reliance on the federal Seasonal Agricultural Worker Program (SAWP) and to a lesser extent, the Temporary Foreign Worker Program (TFWP) for farm and food-processing labour. These federal programs allow employers to hire foreign nationals as temporary or seasonal workers—who we refer to as migrant farmworkers^3^—in positions not filled by Canadians. Typically, between 50,000 and 60,000 foreign agricultural workers come to work in Canada each year through either program, though this number increased to 79,000 between March 2020 and June 2021, corresponding to the initial year of COVID-19 in Canada.^4^ Most of the workers in our study arrived through the SAWP (10 workers), which has facilitated the seasonal recruitment and employment of farmworkers largely from the Caribbean and Mexico since 1966. Two of the workers we interviewed arrived through the agricultural stream of the Temporary Foreign Worker Program.

Temporary forms of labour migration are very common and heavily regulated in the Canadian context, where an ever-expanding migration bureaucracy manages entry, determines the terms of residency, and sets the conditions of labour. Migrant workers can be found in a range of labour markets, but are highly concentrated in service, hospitality, construction, care, and of course, agriculture. In recent years, a growing number of migrants have been filling temporary slots on farm and in adjacent food processing sectors but are recruited through the low-skilled stream of the TFWP, as opposed to the SAWP. As a result, different cohorts of migrants, often engaged in similar kinds of labour, have different rights and, notably, levels of access to permanent residency. The SAWP is one of the few temporary migrant labour programs in Canada that does not facilitate or permit transition to permanent residency or citizenship, which means workers remain dependent on the program over the course of their working lives. Farmworkers who return year after year are “permanently temporary” (Walia 2021). And although it is technically possible to become a permanent resident through the agricultural stream of the TFWP, in practice it is very difficult for farmworkers to meet the conditions required.

Taking Nova Scotia’s local food system as a case study, we find that noncitizen migrant labour is central to small and medium-sized farms, similar to Gray’s (2014) research in the Hudson Valley, New York and Weiler, Otero and Whittman’s (2016) examination of alternative food network farms in British Columbia. Like these studies, we find evidence that “precariousness has become a feature of hired work not only in so-called ‘industrial’ agriculture, but also amidst efforts to realize more socially just and ecologically sound alternatives” (Weiler et al. 2016, p.19). Our research thus contributes to an area of critical food studies that challenges unreflexive celebrations of localism, imagined as conflict-free, environmentally sustainable, socially just alternatives to industrial agriculture, and which tend to ignore labour, local politics, and are prone to corporate branding and cooptation (Alkon and McCullen 2011; Allen et al. 2003; Besky and Brown, 2015; DuPuis and Goodman 2005; Feagan 2007; Gray 2014; Guthman 2004; Hinrichs 2000, 2003). Drawing on this critical approach, we argue that the celebration of local foods by government and industry is a form of “local washing” which obscures their lack of financial and policy commitment to farmers and ignores systemic inequalities. In turn, many farmers contend that sky-rocketing production costs and the lack of “local” (Canadian or permanent resident) workers can only be managed through the recruitment of migrant labour. Migrant labour, in other words, emerges as a cure all for both cost-saving and labour shortages.

After a brief discussion of our research methods, we describe several key features of agriculture in Nova Scotia, including efforts to relocalize food production. We situate our survey findings in the literature on farmers markets and subscription boxes and observe that while some individual consumers are committed to buying and eating locally, they have little insight into the labour that produces that food. The second half of the paper moves from local washing to the deepening reliance on permanently temporary migrant labour. Our interviews with farmers and farmworkers reveal a higher level of expected productivity for migrants compared to resident workers. For migrants this includes higher precarity, vulnerability, and an interdependence between local farms and migrant workers. A central finding is that even where farmers commit to more equitable and compassionate labour relations vis-à-vis their migrant farmworkers, and where consumers are committed to ethical forms of consumption, these dynamics persist. Local washing obfuscates these dynamics and the deepening transnationalization of social relations embedded in Nova Scotia’s local food production.

## Methods

Employing mixed qualitative and quantitative methods, our research examined the perspectives and experiences of farmers, farmworkers, and consumers about local food production and availability with special attention to questions of labour and the federal Temporary Foreign Worker and Seasonal Agricultural Worker Programs. Data collection between 2020 and 2022 was primarily organized around three sets of actors, each integral to local food: (1) farmers who produce for local markets; (2) farmworkers whose labour is required for that production; and (3) consumers who purchase local produce. Additionally, we interviewed 13 key stakeholders across multiple agriculture sectors: nine interviews with farmers’ market organizers and managers and four interviews with agricultural associations and food movement organizations. All interviews were transcribed and analyzed for key themes and information.^5^

### Consumers

We surveyed farmers’ market consumers about local food in two waves. The first wave, conducted entirely online in 2020 due to the COVID-19 pandemic, included 321 shoppers across 40 farmers’ markets, and was posted via social media and sent out in a newsletter from the Farmer’s Markets of Nova Scotia, a non-profit FM cooperative founded in 2004. The second wave, in 2021, captured an additional 43 respondents online and 154 in-person at nine different farmers’ markets located across the province.

### Farmers

Fourteen interviews were conducted with farmers and one farm manager speaking on behalf of the farm on topics of local food, type of farm, agricultural practices, farm labour, and the effects of COVID-19. A further 54 farmers completed an online survey about their labour and recruitment experiences. The survey was circulated by the Nova Scotia Federation of Agriculture and shared on social media and was live from January to March, 2020.

### Farmworkers

In-depth interviews were conducted with 12 migrant farmworkers (10 men and 2 women) from Jamaica and Mexico who were between the ages of 30 and 60. Ten workers were employed through the SAWP and two arrived through the agricultural stream of the TFWP. Interviews were conducted in English and Spanish, by telephone, via Skype or in-person, and focused on the workers’ histories of migration, livelihood strategies, and perspectives on SAWP and local food. Interviews were read and analyzed for information on the federal programs, their workday, leisure time, food production, cooking, family, remittances, racism, abuse, farm inspections, and COVID-19. We also conducted interviews with two Canadian-resident (non-migrant) farmworkers.

## Agriculture in Nova Scotia

In the late 1960s, Canada’s federal government put forth its vision of the future of the country’s agricultural production in a document called “Canadian Agriculture in the Seventies” (Agriculture and Agri-Food Canada 1969). Reinforcing nascent neoliberal ideology, the document forecasted that two-thirds of farmers would need to abandon the sector to ensure its on-going viability. Though met with resistance, the report’s recommendations reflect what has largely come to fruition over the last fifty years: that the nation should prioritize rationally-managed and profit-oriented commercial farms, preferable for their self-sufficiency, productivity, and reduced reliance on state intervention and support (Bryan 2017). Such independence, however, has been mostly rhetorical. As it conglomerated, the Canadian agricultural sector became increasingly dependent on international markets for both food exports and imports (Kissinger 2012). Smaller family farmers faced an income crisis or “a cost-prize squeeze”—that is, when the cost of inputs, manufactured off-farm, are higher than what farmers earn selling their goods (Wiebe 2021, p.163). At the same time, since the mid-1980s, increasingly mechanized and capital-intensive, some Canadian farms have ramped up production but –like their smaller-scale counter-parts—have still suffered an income crisis (Qualman 2011; National Farmers Union (NFU) 2015; Wiebe 2021).

Over this period, Nova Scotia has seen its net farm income decline and farm debts rise, as its rural populations age and younger people out-migrate (Stalker and Phyne 2014). Over the last 100 years, it has lost 90% of its farms, and more recently, between 2016 and 2021 alone, it went from having 3478 farms to 2741 (Statistics Canada 2022b)—the largest decline in the number of farm operators across all provinces in Canada (Nova Scotia Economics Statistics Division 2022). This decline is reflected in the province’s landscape; of the approximately 70% of land that is privately owned in Nova Scotia, at most 30% of that is now designated agricultural land (Devanney and Maynard 2008). Nova Scotian farm operators were the oldest on average nationally in 2017, with 58.8% of farm operators aged 55 years and older (Statistics Canada 2017). The age structure of farming, and the extremely low incidence of farm succession plans, is undoubtedly linked to the difficulty of making a living from farm sales. With the closure of regional vegetable and fruit canneries, poultry and pork processing plants, and the preference of large supermarket chains to source the cheapest products nationally and internationally, some small farmers were left without an accessible market. Smaller food sellers in Nova Scotia face multiple challenges in competing with supermarkets and must raise prices but, in so doing, they risk losing customers. Poor income and sales prospects have demanded that farms find places to cut costs, and farm labour has become the only conceivable place left. Farmers in Nova Scotia, like elsewhere in the country (Basok and Bélanger 2016; Binford 2013; McLaughlin & Hennebry 2013; Weiler et al. 2016), lower their labour costs, and fill an apparent gap in available labour, by turning to the Temporary Foreign Worker and Seasonal Agricultural Worker Programs and hiring highly productive workers whose specialized manual skills are devalued.

Nova Scotia possesses several unique characteristics due to the scale of agricultural production in the province and a rural population of sixty percent. For example, national (and international) trends toward monocultural crop production and agribusiness conglomeration “appear less pronounced” in the province (Andrée et al. 2016, p. 464). This context might support one strategy for small farms looking to stay profitable: the niche, mixed, explicitly small, and local farm. Thus, most newer farms are smaller, and target niche or speciality markets.^6^ In their study, Andrée et al. (2016) note that “entrepreneurialism in local and niche markets appears to be vitally important as an enabler to the future success of Nova Scotia’s agricultural communities and in many cases supports more sustainable forms of agriculture” (2016, p. 465). Indeed, the average size of a NS farm is 263 acres, compared to the average in Canada of 809 acres (Government of Nova Scotia 2022). Importantly for efforts to “re-localize” food, Nova Scotia also has a significant history of local food production and alternative food initiatives.

Much like in the rest of Canada, First Nations livelihoods and systems of food production in Nova Scotia were undermined with colonization and the expansion of settler society (Morrison 2011). In Atlantic Canada, the British Crown entered into Peace and Friendship Treaties with the Mi’kmaq, Wolastoqiyik and Passamaquoddy, that set out mutual obligations between the Crown and Indigenous peoples, but the recognition and exercise of treaty rights has been a contentious, litigious process. In 1999, the Supreme Court of Canada formally recognized the right of Indigenous people in the region to hunt and fish for a “moderate livelihood” in a landmark case called the Marshall decision (McMillan and Prosper 2016).^7^ Another important struggle in the province revolves around African Nova Scotians’ on-going efforts to seek legal title to land given to Black Loyalists and refugees of the war of 1812, in recognition of their support during the American revolutionary war. Because the government gave the land without official deeds, many descendants of these Black settlers have been living on and working land they did not officially own for generations, while often paying property taxes. In 2017, the province finally approved funding to help residents in five historically Black communities gain legal ownership to their own land (Borden Colley 2017). Although we focus on direct agricultural markets as an avenue for re-localizing food, it is important to point out that long-standing Black and Indigenous struggles to have legal title to their land or exercise treaty rights to fish, hunt, and gather are part of such efforts in the province.

## Re-localizing food

During the 1970s, despite the overall trajectory in Nova Scotia toward farm consolidation, the province became a destination for counter-cultural ‘back-to-the-landers’ who pursued alternative, small-scale agriculture, including organic (Hetherington 2005). Today, the province is home to the country’s first fair trade town in Wolfville, the highest number of farmers’ markets per capita in Canada, and a robust set of Community Supported Agriculture (CSA) programs (Spice 2012; Halifax Food Policy Alliance 2014). Direct agricultural markets, such as farm gate stands and farmers’ markets, long pre-date the rise of grocery stores and supermarkets as the main venue for purchasing food. Currently, Nova Scotia hosts around 69 farmers’ markets (Farmers’ Markets of Nova Scotia, personal communication). Following a period of decline nationally between 1916 and 1970, associated with the rise of industrial foods and grocery stores, farmers’ markets underwent a revival in the 1970s in response to environmental concerns and government support (Basil 2012). Farmers’ markets and various CSA programs in Nova Scotia have proliferated in more recent years, leading some to conclude that the alternative food initiatives in the province have been, on balance, successful in “foster[ing] the development of new projects and opportunities for businesses, farmers, and some fish harvesters” (Andrée et al. 2016, p. 473).

In general, direct market venues, CSAs and subscription produce boxes aim to lessen the role of the middlemen and provide greater return to producers. Farmers’ markets are often posited as a cornerstone of efforts to relocalize food, both in academic and grey literature, because they: (1) make local food products and producers regularly visible in public settings, (2) enable some producers to diversify their enterprises, (3) incubate small businesses that may use non-conventional production methods, such as no-spray (of chemical pesticides) or organic and (4) create spaces for both market transactions and nonmarket social interactions (Gillespie et al. 2007, p. 66). In comparison to supermarkets, farmers’ markets, and local food systems generally, often require less food packaging, and storage capacity, offer less-intensively grown agricultural products, and produce less waste (Benis and Ferrão 2017; Feenstra 1997; Hinrichs and Lyson 2006). Direct agricultural markets can also sell some visually imperfect produce that would not meet the standards of wholesalers and supermarket chains, which is one way of reducing food waste and, in turn, costs.

Research on direct agricultural markets explores the extent to which they are influenced by values other than purely economic ones, or the nature and degree of their social “embeddedness.” Although *all* economic exchanges and markets are also cultural practices mediated by social relations, norms, and values—even the behaviour and motivation of idealized rational economic actors (Carrier 2018)— the concept of embeddedness is insightfully used by Hinrichs (2000) to argue that direct agricultural markets can entail contradictory social relations and dynamics of power and privilege. Some direct agricultural markets are more socially embedded than others: when compared to exchanges at grocery stores, supermarkets and even at farmers’ markets, CSAs and subscription boxes are are lower on a scale of “marketness”—that is, for subscribers they are exchanges that are less price-driven or guided by economic goals (Block 1990; Hinrichs 2000). The up-front commitment entailed in CSA and subscription boxes “symbolizes members' shared acceptance of the risks farmers assume in farming and their willingness to subordinate their own economic interests, if need be, to support the [farmer]” (Hinrichs 2000, p. 300; Block 1990).

The commitment runs both ways, however. Many farms in Nova Scotia that participate in subscription boxes and farmers’ markets write newsletters, provide recipe suggestions for their customers, and host children’s activities like corn mazes and seasonal farm festivals. These activities may encourage a better understanding among consumers of farmers’ circumstances, even perhaps building social connections and community, but the task of maintaining community connection often falls to farmers. The burden of a “shared community” is unevenly distributed (Hinrichs 2000, p. 300).

Compared to the opaque, distant, and anonymous relations between producers and consumers in longer food chains, relations in direct agricultural markets tend to be more personal, face-to-face interactions, enacted in a shared space. Shoppers look to farmers’ markets for social interactions, community building, a consumer-producer bond, and as a way of supporting local producers and the local economy (Gale 1997; Novak 1998; Cummings et al. 1999; Jablow and Horne 1999; Feagan et al. 2004; Feagan 2007). However, according to previous studies, the number one reason patrons buy food from direct agricultural markets or from local food systems, is its perceived high quality and freshness compared to imported food found in grocery stores and supermarkets (Sommer 1980; Govindasamy et al. 1998; Novak 1998; Cummings et al. 1999; Holloway and Kneafsey 2000; Brown 2002; Sanderson et al. 2005). Direct agricultural markets may be perceived as viable alternatives to homogenous global (industrial) food, in addition to offering higher quality food, and a sense of local distinctiveness, naturalness, artisanal production, and traceability (Kirwan 2004, 395). Shopping local can be an enactment of “ethical consumption” as the result of reflection about the potential impact of one’s purchases and practices, even if inconsistently practiced or reasoned. In a study with FM customers in British Columbia, researchers found the idea of “good food” encapsulated the values about what people think is good for themselves, for their community, and for society, which included a temporal element – responsibility toward future generations (Connell et al. 2008, p. 170). FM consumers tend to focus these qualities over price, and while they still expect good value for money (Brown 2002), the ability to deprioritize cost is one reason why FM patrons are often predominantly middle-class (Brown 2002).

Farmers’ markets and CSAs provide a valuable alternative to the corporate food system and supermarket chains, as well as a sense of social connection and trust between shoppers and farmers, which are the hallmark, and comparative advantage, of direct agricultural markets. However, they “do not challenge the fundamental commodification of food”, nor do they necessarily make price irrelevant for consumers or farmers (Hinrichs 2000). Indeed, some studies found that one reason farmers participate is because of the premium price they can ask at a farmers’ market compared to what they receive from products sold via wholesale chains that end up at the larger grocery stores (Hinrichs 2000; Kirwan 2004).

When discussing the reasons why they participate in farmers’ markets, our participants –shoppers, managers, and farmer interviewees and survey respondents– all articulated the importance of values other than, or in addition to, economic considerations, including how they foster social interactions and ties, a consumer-producer bond, a reduction in food miles, and the freshness and quality of the food. In our surveys, FM consumers indicated that they felt it was important to support local farms (see Ellsworth et al. 2020). They also felt that local food is healthier and fresher, expressed a desire to see and meet the people who grow one’s food and a commitment to reducing environmental impacts by supporting lower impact agriculture or pesticide free crops, and buying food that travels less. These responses were not overly surprising and fit in the spectrum of motivating factors for shopping at farmers’ markets or locally as noted in other studies.

The economic bottom-line was, of course, an important consideration for the farmers we interviewed and surveyed. One Nova Scotia farmer we spoke with noted that farmers often struggle with pricing products fairly for both themselves and consumers, explaining that farmers are often “price takers, not price setters.” Several other farmers also said they are constrained by the prices that are laid out by larger supermarket chains in the region, and by the perception that consumers will not pay *too* high a premium for local products. Other farmers who undertook a cost analysis on their business and increased prices said while they have lost a few customers, it was not as many as they had expected. As farmers told us, this delicate dance of price-setting is intrinsically connected to the cost of labour:

The bottom line in all these agricultural problems is dollars. If the food was a price that supported a proper local business we could pay [our farmworkers] more, more benefits and healthcare. We could afford to do all that stuff society expects, and maybe rightfully expects. But the thing is, it’s hypocritical, they want their farmers to live based on a price that’s based on abuse of the environment or people somewhere else, and that’s the bottom line (Farmer interview 2020).

Though not always the case, FM prices are sometimes perceived as higher than supermarket chains in Nova Scotia. This, coupled with the additional time and effort it might take when compared to the one-stop shopping of supermarkets, make farmers’ markets unattractive or inaccessible to some consumers. Additionally, as spaces, farmers’ markets can express a community imaginary of predominantly affluent white consumerism, “ethical posturing” (Guthman 2007b) and a “defensive localism” that discourages lower-income and racialized people from participating (Alkon & McCullen 2011; Feagan et al. 2004, p. 240; Guthman 2008a and 2008b).

There are efforts among farmers and farmers’ markets in Nova Scotia, however, to make local foods accessible to lower income residents. Acting on values in addition to price and profit, several farmer interviewees mentioned donating vegetable boxes to food insecure households and inviting residents to glean on-farm to provide free food while also reducing food waste. Along similar lines, the Farmers’ Markets of Nova Scotia Cooperative developed the Nourishing Communities Food Coupon Program in 2019, funded in large part by the Nova Scotia Government, which provides “food bucks” redeemable at participating markets to make local food more accessible to households experiencing food insecurity (Farmers’ Markets of Nova Scotia 2021). Additionally, several farmers’ markets have developed programs to limit their environmental impacts: the Wolfville Farmers’ Market implemented a Zero Waste Initiative that aims to eliminate single-use items as much as possible (Lang 2019).

Our research found that farmers and shoppers who participate in subscription boxes and farmers’ markets are motivated by values and considerations in addition to economic calculations of price or profit; however, there are tensions between the expressed values and goals of the participants in direct agricultural markets and the contradictory social relations they embody. These contradictions and tensions are particularly pronounced when we consider the centrality of permanently temporary migrant labour in the production of local foods, discussed further below, and the little our respondents knew about labour recruitment practices and working conditions on the farms producing their food (Ellsworth et al. 2020).

## Celebrating, not supporting, local in Nova Scotia

The Nova Scotia government supports numerous initiatives to foster local agriculture and food consumption, often in collaboration with community and sectoral organizations. Noteworthy examples include: a mobile food market bus that brings local produce at affordable prices to several lower income and racialized communities since 2016; the *Taste of Nova Scotia* program and marketing campaign^8^ promoting Nova Scotian food to domestic and international tourists; the Food Miles’ Project led by the Ecology Action Centre and the Nova Scotia Federation of Agriculture; the highly influential Ivany Report^9^which set goals for provincial food production and consumption; and the province’s 2007 Environmental Goals and Sustainable Prosperity Act, amended in 2012, which also established targets for increasing local consumption to 20% by 2020 (EGSPA 2012). While a full explanation of these initiatives and policies is beyond the scope of this article, we note that, as of yet, this consumption target has not been reached^10^ and there is a need for greater government support for small-scale environmentally sustainable agriculture (see also Bibeau 2020).

In 2022, the provincial government launched a consumer reward program and media campaign, *Nova Scotia Loyal*, to help boost local food to 20 percent of all food purchases in the province by 2030,^11^ but without clear insight into program effectiveness nor into how best to measure the current level of local food consumption (Laroche 2022; Moscovitch 2022). Accompanied with enticing marketing campaigns promoting local restaurants and Nova Scotian food culture, programs such as *Taste Nova Scotia* and *Nova Scotia Loyal* are, we argue, forms of government and industry “local washing”. They celebrate and fetishize the local, often commodifying it for tourists to the region, while providing, according to farmers, little meaningful support for relocalization efforts, climate-forward agriculture, or an alternative to a deepening reliance on migrant labour. The marketing of these programs obscures the reality that the province has not achieved the goals it set for increasing local food production and consumption, decreasing the per capita waste disposal rate, nor for developing and implementing a strategy to expand the green economy in which agriculture must play a role (Nova Scotia Environment 2017).^12^ Trading on facile and incomplete accounts of the benefits of local consumption, campaigns like these capitalize on public anxieties concerning climate crisis and global food supply chains, rather than committing to redressing their underlying causes. At the same time, they responsibilize individual consumers for what is likely only achievable through large-scale systemic change.

Moreover, the image of “local” used in buy local campaigns rarely, if ever, shows the central role of migrant labour. As Evelyn Encalada Grez explains, “food campaigns […] urge Canadians to buy local but do not include the images and voices of racialized migrant farm workers toiling in the farms and fields” (2018, p. 20). Several studies in the United States and Canada have examined the role of precarious im/migrant labour in local, smaller-scale agriculture and noted their absence in discourses about local, family farms as a more socially just and ecologically sound alternative to the industrial food system (for eg. Encalada Grez 2018; Gray 2014; Guthman 2004; Weiler et al., 2016). In her research on small-scale local farms in the Hudson Valley of New York, Margaret Gray (2014) found that as valley farmers faced financial strain, they worked with state bureaucrats to recruit noncitizen migrant labour. Yet, academic and popular writing on small-scale, sustainable agriculture, tends to present “an idealized, agrarian vision of soil-and-toil harmony on family farms” overlooking their reliance on the noncitizen labour of vulnerable undocumented migrants and guest workers (2014, p. 9). Along similar lines, Julie Guthman’s earlier research in California found a disjuncture between the promotion of organic agriculture as a part of a rural “renaissance” or new agrarianism that equated “both social justice and ecological sustainability with small-scale family farming” (2004, p. 12) on the one hand, and the realities of paid labour on-farm, in warehouses, and at markets, on the other.

Farmers in Nova Scotia receive a smaller percentage of the spending that households devote to food each year. A 2010 report by the Ecology Action Centre—a local environmental policy advocacy organization—and the Nova Scotia Federation of Agriculture emphasized a need to relocalize the food system in the province as they noted that despite a considerable amount of local production, a “surprisingly small proportion of the vegetables we eat in Nova Scotia are actually grown here” (Scott and MacLeod 2010, p. 14). The report examined whether Nova Scotians were eating local, and if not, where their food was coming from. It found that the average distance traveled by foods in the National Nutritious Food Basket from its origin to Halifax, Nova Scotia was 3,976 km, “not [including] farm inputs or additional kilometres for warehousing or shopping trips” (Scott and MacLeod 2010, p. 4).^13^ At the national level, around 30% of agricultural and food commodities consumed in Canada are imported, and the highest food miles related to emissions were fruits and vegetables which arrive by truck from the US and Mexico or airfreight from overseas (Kissinger 2012). However, the distance food travels does not provide the complete picture of the carbon emissions in food production and distribution (Brunori et al., 2016; Coley et al. 2011). Even seemingly “local” food chains often combine local, regional, and global elements, along with different relations of production, making clear distinctions difficult to delineate. Indeed, using only physical distance criteria, as in the case of food miles, can be misleading, as it relies on a single criteria and obscures the extent to which ostensibly local producers may actually engage in a range of production and distribution practices,^14^ such as importing seasonal farmworkers.

We found in interviews and our online farmer survey that certain farm inputs (feeds, fertilizers and so on) are not manufactured nor available locally. Just under a quarter of the 54 surveyed farms said they used mostly or all locally-sourced inputs in production. Moreover, even those who exclusively grow *and* sell locally (producing only for local markets and eschewing export) still rely heavily on non-local production inputs. As one farmer engaged solely in local sales recounted “the plastic bins we use, the packaging and labels, the equipment, we try to reuse as much as we can and source as close to home as possible, but none of it is manufactured locally; the closest we might get is, like, Quebec” (interview 2021). In another interview, we heard about one berry farmer who had, for many years, been making his own wooden pint boxes out of poplar growing on the farm property, only to be told by a large grocery chain that they would only carry his fruit if he switched to plastic clamshell packaging.

In this vein, farmers spoke to us about a lack of infrastructure and meaningful support for smaller or medium-sized producers and alternative agriculture that would help localize production and consumption in the province. Some of the farmer interviewees identified ways to better support local food producers. For instance, improving SAWP to better compensate and protect workers, and provide a path to permanent residency or citizenship was mentioned. One farmer suggested that public institutions like hospitals and schools could provide healthy, fresh meals using local produce; that the province could make a commitment to regular, large orders from farmers in the region; that perhaps government could restrict imports to give local products a better chance. In the absence of an infrastructure that adequately and flexibly supports small and medium-scale local producers, farmers spend time applying for small programs and special funds for discrete needs (e.g., installing a pond on the farm). One farmer suggested that government paperwork to apply for programs and funds at the very least be more streamlined and user friendly. In sum, the conditions are not in place to better support the localization of food production and consumption in the province. This is compounded by what farmers perceive to be a lack of locally available labour.

### “Work harder, faster, longer”: The local reliance on migrant farmworkers﻿

Existing research on SAWP is excellent but has not fully examined the program in the Atlantic Region. This is largely a matter of numbers; most migrants who work on farms are employed in Ontario (40%), Quebec (32%), and British Columbia (18%) with only 2.6% in Nova Scotia (Employment and Social Development Canada 2020). That said, despite this relatively low number, the province has seen a considerable increase in recent years. In 2010, 957 positions were approved in Nova Scotia under both the SAWP and the primary agricultural stream of the TFWP (Statistics Canada 2018). In 2021, this number increased to 2,412 positions (Statistics Canada 2022a).

Researchers and advocates have importantly shown that SAWP contributes to the systemic, legal and normalized production of a range of less than full citizenship statuses (Goldring et al. 2009, p. 243). The program renders workers vulnerable to exploitative, unsafe, unhealthy, and potentially coercive situations as workers’ work permits are valid with only a single, designated employer. Guest worker programs are designed to provide access to cheap labour while simultaneously placating anti-immigrant and racist sentiments, creating the ‘ideal’ immigrant who is ineligible for state support, and while vital to entire economic sectors, is temporary (Hahamovitch 2003; Barber 2008; McLaughlin 2010). As previously mentioned, the SAWP is one of the few temporary migrant labour programs in Canada that does not facilitate or permit transition to permanent residency or citizenship, which means workers are permanently temporary, often returning year after year.

The federal programs are regarded as a win–win situation by the governments of both sending and receiving countries because they appear to redress unemployment in the global south by employing people in the global north, where ostensibly there are labour shortages. More accurately, and as revealed in our interviews with farmers, these shortages are in people *willing* to work for around minimum wage at jobs that are demanding, physically taxing and sometimes repetitive.

Each farmer we interviewed, and 33 out of the 54 who responded to the survey, reported difficulty in recruiting and retaining workers. In the absence of other options, some of these farmers relied on the federal Seasonal Agricultural Worker Program (SAWP) and to a lesser extent, the agricultural stream of the Temporary Foreign Worker Program (TFWP). As one farmer we interviewed explained: “we used to get people all from Atlantic Canada come in and pick [produce] and you know, have retired people. And now we just aren’t getting enough local workers” (interview 2021). Another challenge noted by one farmer interviewee was the seasonal nature of farm work. Explaining that with the short season, it was difficult to find and hire, for example, an experienced forklift operator, he said “trying to find people with that experience who want to work for three months. It's very challenging” (interview 2021). Some of this difficulty is due to the aging population in rural Nova Scotia, and the fact that a substantial proportion of younger, less educated workers go west in search of high-wage employment in Alberta’s oilfields (Foster & Main 2018).

In addition to changing demographics in the region, a number of farmers attributed their inability to recruit locally to inadequate skill sets and attitudes amongst local workforces. Farmers who responded to the study’s survey said they needed, above all else, “hard workers”—people who will be committed to doing difficult, physical labour. As another farmer explained: “We have an aging workforce in Nova Scotia and a migration of youth out of the province. Honestly people do not love working hours in the sun when it’s 30 degrees [Celsius] picking [fruit]. It was one of those things where we really [needed] the skilled labour” (interview 2021). Though farmers in the interviews and surveys would make vague reference to a lack of skills, specific skills were not as commonly mentioned, nor seemingly as important, as simply having the right attitude and work ethic. While some employers said the right workers are less plentiful in local labour markets, others admitted that they were simply unable to pay what local workers with the “right attitude and work ethic” were worth. Here, we encounter an important insight, often acknowledged by farmers, concerning differently positioned and stratified workers, expectations around rates of pay for particular kinds of work, and the food system that structures each.

When asked how to address the shortage of farm labour, one farmer responded: “the problem is, a lot of these jobs are minimum wage jobs and as long as society is demanding cheap food, you’re demanding low wages on farms. And that’s really our problem, so we can talk about how to attract more people to the farm. But how do you attract people who are going stay seven or eight months and keep it up?” (interview 2020). Still others felt that low wages were only part of the problem. Here, again, the perception that local workers are disinclined toward hard work informs recruitment efforts. For example, reflecting on why they had waited to recruit through SAWP, and what prompted the decision to do so eventually, another farmer explained:

[we waited] “based on the fact that you feel loyalty to your community, you don’t want people to say mean things, which they will, about taking away people’s jobs and all that kind of stuff. But the reality is: as years went by, we are finding it more and more difficult to get people to weed and harvest crops. So in 2006 […] we just couldn’t get anybody to come out and weed anymore. That was it. Everybody was done. So at that time I think minimum wage was around $7.50 an hour, right around there [and] we determined it would take 45 minutes to weed a row, so we figured we allow people an hour to weed a row [and we’d pay $20 a row]. So, they’re going to make really good money and we figure that’s the only way we’re going to save our crop and we had… it was just over 20 people that came out over like a three or four or five day period, we advertised at the time that we needed this help and I think the longest a person lasted was like 15 minutes” (interview 2020).

In a similar vein, a different farmer said the issue is not the pay: “If we didn’t have foreign workers, I don’t think our farm would survive. I don’t think it matters how much you pay. There’s just not that pool anymore that wants to do [the] work” (interview 2021). Another farmer interviewed said the program is “the best source of good reliable help for sure. It’s cumbersome from the way the government does it and every year it gets worse” (interview 2020). He continued:

“[the government goes] out of their way to make it painful I think, you know we’ve been told point blank – you know, we do not want you to hire these guys, you need to hire Canadians, is what they’ll tell us and they say this is a premium program. In other words, it’s made to cost you more than hiring local. But it makes no difference with the locals, [who] just don’t show up no matter…they’re always like ‘oh, if you paid me 50 bucks an hour I’d come every day’. Yeah, I betcha. He still wouldn’t do anything. So, these [migrant worker] guys cost us more, but they are that much better, they’re more than worth it. Not to mention we have to post job ads every year anyway. And we, you know literally get maybe one, one application [from Canadian residents] every second year, it’s all we get from people applying. Nobody applies.”

In our interviews with migrant farmworkers, all twelve said they recommended the SAWP to their family members and friends, when asked, mentioning the lack of employment options or difficulties with farming in Jamaica or Mexico. As the literature demonstrates, and our interviews reinforced, farmworker wages in Canada provide needed economic support – “a lifeline to their households back home” (Binford 2013; Wells et al. 2014, p. 151). Farmworker recommendations of SAWP to friends and family, and any positive comments about the program, must be seen in relation to its disciplinary nature: SAWP farmworkers are reliant on a single designated employer who can send them home or aid in blacklisting them. The point is not that all farmers in the province would do this, but rather that they *could*.

Every migrant worker interviewee told us they would like the option to live in Canada—although two who had relatives in other parts of the country said they likely wouldn’t stay in Nova Scotia. When we asked what their experiences with the program were, one Jamaican worker who had been coming to the province since 2010, said, “Well, I can say it’s very hard but at the end of the day, it works. Nothing comes easy” (interview 2020). The same farmworker expressed disappointment at not having a pathway to residency: “I want the opportunity but it’s not coming to me. […] I think they should look into it and give us some form of status.” Not having a pathway to residency or citizenship was consistently mentioned as a negative aspect of the SAWP. Other difficulties discussed were being away from family, feeling isolated on the farms from towns or social activities, encountering racism in the wider community, long hours, and mistreatment on-farm.

Two of our migrant interviewees mentioned preferring the work in Nova Scotia compared to Ontario or British Columbia, and that they felt they were treated better in their current jobs. The smaller scale of Nova Scotia farms compared to these other provinces may provide a somewhat more positive day-to-day experience for some workers. In contrast, one farmworker interviewee complained about a previous employer who had imposed a curfew on farmworkers to be back in their accommodations, provided inadequate mattresses covered in mouse droppings, and started to hire Mexican workers after Jamaican workers complained about their living and working conditions (interview 2022). He explained that the Jamaican employees “banded together in a way and, you know, had a conversation with the boss…and there’s twenty of them that didn’t get brought back. [So they brought in] Mexicans instead of Jamaicans” (interview 2022). This supports Binford and Preibisch’s (2013) findings that Canadian employers are able to “country surf” looking for the hardest working labour force and those workers perceived to be less likely to complain. One key difference between Mexican and Jamaican workers, is that the former may not be able to communicate in English and are less likely to have family living in Canada. Mexican and Caribbean workers are racialized by the Canadian government and employers, but in different ways, as being better at –or showing a preference for– certain on-farm tasks and more or less likely –or able–to complain.

Farmworkers work longer hours than the legislated full-time workweek in Nova Scotia—our interviewees said a typical workday on farm is 10 or 11 hours (interview 2020). One farmworker put it to us this way: migrant farmworkers “work harder, faster, longer” than their Canadian counterparts (interview 2022). This aligns with other research showing that the SAWP is structured to demand higher levels of productivity from workers than is expected from local residents (Basok 2002; Binford 2004). As workers are tied to a single designated employer, the program generates *coerced* productivity (Basok and Bélanger 2016). And farmers do see and reap the benefits: one Nova Scotia farmer told us that a “Mexican worker was worth three Nova Scotians” (interview 2020).^15^ Narratives about Mexicans as “hard workers” or as having “good attitudes” may contribute to a sense of self-worth for some workers –such as those interviewees who spoke with pride about being hard workers—but they are also tropes that naturalize exploitation as a cultural work ethic; they naturalize exploitation in the sense that workers face the possibility of deportation and loss of income if they complain or report unfair wages and dangerous work conditions, a precarious position both SAWP workers and undocumented workers share in North America (Fitting 2016; see Gomberg-Muñoz 2011). Between 2009 and 2018, temporary foreign workers launched thousands of complaints with the Mexican government about Canadian employers. In that time, “89 complaints [were] connected to Nova Scotia farms”, including being forced to work without pay (Grant 2019).

The COVID-19 pandemic brought some of the plight induced by this structured precarity to national attention. There were many news media stories on the program and a range of difficulties faced by workers, particularly in larger provinces like Ontario. In the summer of 2020, migrant workers at one Nova Scotia farm lodged complaints with Migrant Workers Alliance for Change, claiming that the farm provided unsafe working and living conditions, and that they were not paid for their mandatory quarantine period and were told to keep quiet when government agents visited the farm for inspections (Ziafati 2020). If they did not comply, they faced deportation. The farm denied the claims, but 40 different workers contacted the Alliance and were backed up by residents. As this story broke, the Canadian federal government announced a $58.6-million investment into the temporary foreign worker program to safeguard the health and safety workers from COVID-19.^16^ By centering COVID-19 in the funding package, the government framed the situation as a symptom of the pandemic.

Migrant farmworker interviewees suggested several ways to improve the SAWP: not surprisingly, first and foremost was providing workers a pathway to permanent residency and citizenship. Along similar lines, one SAWP interviewee wanted the ability to visit family elsewhere in Canada and the United States, while another mentioned the ability to come and go from Canada without being tied to an individual employer. Several farmworkers thought that their wages should be higher, especially those on farms that paid only the provincial minimum wage, and that they should have access to the same government benefits that Canadian workers have, such as employment insurance and programs supporting parents with young children, that they pay into or their tax payments support. An interviewee who had investigated ways to pursue citizenship expressed concerns about their English given the requirement to pass a language test. Farmworkers also articulated the need for better protections from those farmers who are abusive or neglectful. As one interviewee put it, the liaison staff hired by Mexico and Jamaica ostensibly to help their compatriots in the program, need to visit farms and better support workers. As it is, the liaison staff act as though they “are there [to help] the farmers. Most of them are there for the farmers, they’re not there for the workers” (interview 2022). Finally, one Jamaican farmworker suggested raising awareness or educating Canadians about the people who grow their food. He continued by saying, “I would hope that […] Canadians would recognize that we’re here to work, and not only to benefit, but in order to help to feed persons” (interview 2022).

## Conclusion

Much of the research on direct agricultural markets and local food examines the nature of the relationships and the values embodied in exchanges between farmer and consumer. These exchanges are seen to offer alternatives to the conventional food system that can potentially nurture social ties and community networks, enhance consumer knowledge about where their food comes from, and provide more environmentally sustainable food by requiring fewer travel miles and incubating initiatives for lower input agriculture. While the locality of food alone does not determine its environmental sustainability, there are correlations with reducing food waste and promoting ecologically sound agricultural practices; for example, locally produced food sold at farmers’ markets and subscription boxes requires less food packaging and storage requirements as it can be picked and sold when ripe. Farmers’ markets also often engage in waste reduction, zero-waste, and other environmentally focused projects and act as a hub for less-intensively grown agricultural products.

Our research contributes to this literature by foregrounding the increasingly central role that permanently temporary migrant farmworkers play in the production of local foods in Nova Scotia, including on farms participating in subscription boxes and farmers’ markets. Although provincial policies contain goals for strengthening local food production and consumption, these goals have not been met. Furthermore, according to farmers we spoke to there needs to be more direct initiatives to support small-scale and sustainable agriculture beyond marketing campaigns and goals. Thus, we argue that marketing campaigns and reward program celebrating local food by government and industry are “local washing.” These campaigns are not matched by financial and policy supports directed at strengthening provincial farms nor address the causes for an increase in farm reliance on migrant farmworkers. Moreover, during the COVID-19 pandemic, the federal government framed migrant farmworkers as essential workers yet did little to address the central problems of the federal programs. The federal government did open a line of funding for farmers to better follow public health precautions during the pandemic, but unfortunately, in some cases, on-farm conditions remained conducive to outbreaks. Tragically, during the pandemic, farmworkers in other provinces have died (Braum and Grant 2020; Levitz 2020).

The consumers we surveyed, whose purchases were motivated by ethical values or ideas of “good” food, had little to no insight into the labour required to grow that food, the conditions of that labour, or what motivates labour migration in the first place. Although discussions of ethical consumption can overemphasize the agency of individuals and overlook structural inequalities in production, Goodman and DuPuis (2002) remind us to avoid reductionist understanding of consumers as passive agents, who are unable to see beyond the commodity fetish. Examining the transformative possibilities of both production and consumption is part of an approach that “sees the political possibilities of consumption as less than the revolutionary overthrow of capitalism but more than merely a niche marketing opportunity” (2002, p. 18). They clarify that “one has to see the mutual constitution of social relationships between producer and consumer, and the ways in which market and non-market activities are continually embedded within each other, rather than being contained in separate spheres.” (2002, p. 13). In Nova Scotia, extant research has proposed that ethical consumption can be a collective endeavour in certain communities and can thus be “more than individual desire and economic calculation; it can also be a platform for collective action, though its social movement potential can be debated and challenged (Spice, 2012, p. 10). In other words, if people can move beyond the ethical consumption designations as more than just branding, localization and other alternative food initiative models can still offer an effective avenue for solidarity; we propose that this solidarity *can and must* include labour.

But bringing labour into the localism fold presents new challenges to alternative food movements. For instance, well-known food writer Michael Pollan has argued that if consumers could see who is producing their food and how it is produced, they would change their consumption practices (Guthman 2008a; Reichman 2014), though as Fitting points out, the circulation of powerful racist discourses often directed at racialized migrant workers in North America means that, for some consumers, “knowing who produces and prepares food, might not always generate change in and of itself” (2016, p. 97). Moreover, Julie Guthman and others suggest that the culture of the “personal audit” around food is a troubling symptom of the breakdown of state regulatory power under neoliberalism. The governmental oversight of food, labour, and the environment, in many cases, has either been co-opted by large corporations or made moot by global supply chains that are beyond the scope of any public regulatory agency (Guthman, J. 2007b, p. 263; Guthman, 2007; Reichman 2008, 2014). Guthman J. (2007b) suggests that “food biographies” –like those found in local food marketing campaigns – “eschew a complex engagement with political economic analysis in favor of simplistic ‘farm-to-table’ stories” (in Reichman 2014, p. 170). In a similar fashion, we suggest that marketing campaigns and reward programs celebrating local food in Nova Scotia downplay the very complex connections between local and global food systems, and arguably overstate the benefits of local foods as ethically superior, environmentally sustainable products.

In sum, any real transformation of the food system requires multi-dimensional solutions at home, at the national level, and abroad. It must include practices and policies that support non-conventional, smaller-scale, and more ecologically sustainable farms both regionally and in the global south, improve food workers’ rights, reform immigration policies, and challenge racism. We have emphasized the importance of attending to labour as a blind spot in consumer perceptions of local food and sustainability. Farm work needs to be secure and attractive to local workers; migrant farmworkers need protections against exploitation and arbitrary dismissal, as well as pathways to citizenship; the remaining agricultural land needs to be protected against development and ecological destruction; and farmers need to earn a sufficient income for themselves and their employees.

## Footnotes

1 Following common usage, we use “local” interchangeably with “provincial” in this paper, as Nova Scotia is a relatively small province by Canadian standards (with an area of 55, 284 km2, about the size of Croatia or Togo, and a population of one million in 2022).

2 The provincial government is unsure of how much local food is purchased in Nova Scotia but estimates between 7 and 15% of all purchased food in the province is produced locally (Laroche 2022). Scott and MacLeod (2010) found a decline in percentage of household food budgets spent on local food from 1997.

3 Since we focus on the employer-employee relationship in this article, we refer to farm owners/operators as farmers and their employees as farmworkers, although, of course, they are also “farmers.”

4 From March 2020 to June 2021, more than 79,000 workers arrived to work in Canada’s agricultural sector (Office of the Auditor General 2021). The vast majority of these workers were employed in crop production (91.6%), with 43.8% coming from Mexico, 23.3% from Guatemala and 14.1% from Jamaica. SAWP is the most used program by agricultural producers (Statistics Canada 2021; Statistics Canada 2022c).

5 This study received approval from Dalhousie University’s Research Ethics Board, in accordance with Canada’s Tri-council Policy Statement on Ethical Conduct for Research Involving Humans. All participants were informed about the project, its objectives, any risks association with participation, and the study’s dissemination plan. Participants reviewed consent forms and formally agreed to participate. Any names have been changed and no identifying information is reported.

6 “The proportion of farms producing organic products in Nova Scotia edged up from 1.5% in 2011 to 1.7% in 2016. Nationally, farms producing organic products accounted for 2.2% of the total farms in 2016” (Statistics Canada 2017).

7 In the Marshall decision, the Supreme Court upheld the right for First Nations to fish, hunt and gather in pursuit of a moderate livelihood, after Donald Marshall Jr. defended his right to catch and sell fish.

8 Taste of Nova Scotia. https://tasteofnovascotia.com/about/ (Accessed March, 2021).

9 The Nova Scotia Commission on Building Our New Economy (2014) was chaired by Ray Ivany and is commonly known as The Ivany Report. It was updated by the ONE Nova Scotia collective (2021).

10 One estimate posited that 11.5% of annual household food budgets in Nova Scotia went to farmers within the province, down from 17% in 1997 (Scott and MacLeod 2010). ONE Nova Scotia reports that the consumption of locally farmed, processed, and prepared food has increased since 2014 but largely due to restaurant meals. Consumption of locally sourced food remains below government targets (ONE Nova Scotia 2021).

11 In 2021, the Environmental Goals and Climate Change Reduction Act changed the timeline to achieve the goal of reaching 20% local food consumption to 2030, with plans to set strategies to create a process for measuring local food consumption, improving access to locally produced food, as well as increasing food security and economic growth in Nova Scotia rural communities (Nova Scotia Government, 2022). It also includes goals to reduce greenhouse gas emissions, grow green economies, and improve the health and sustainability of Nova Scotia’s environment.

12 In 2021, a new Environmental Goals and Climate Change Reduction Act includes goals to grow a green economy (Nova Scotia Government 2021).

13 “In order to calculate the distance food is traveling, we chose to use the National Nutritious Food Basket (NNFB) tool. The NNFB contains 66 food items, from 11 different food groupings which reflect the eating habits of Canadians, as well, these foods, in appropriate combinations and amounts, were designed to meet the nutritional needs of Canadians according to the 1992 Canada Food Guide” (Scott and MacLeod 2010, 12).

14 In some cases, imported food can generate lower greenhouse gas emissions when compared to the production of local food, if the local food was produced and distributed by truck in smaller quantities, for example (Coley et al. 2009; Coley et al. 2011; Mundler and Rumpus 2012; Reynolds et al. 2017; Wallgren 2006).

15 Farmers elsewhere in Canada have been quoted saying similar things; for example, “it takes more than two Quebecers to equal the work of a Mexican” (Valiante 2020).

16 The funds included $7.4 million for “increasing supports” to temporary foreign workers ($6 million of that for outreach to workers delivered through migrant worker support organizations), and $16.2 million to “strengthening the employer inspections regime” such as making improvements to how claims of employer non-compliance are addressed. The final $35 million dedicated to infrastructure, addressing health and safety on farms such as employee living quarters to prevent and respond to the spread of COVID-19 (including temporary or emergency housing, PPE and sanitary stations).
